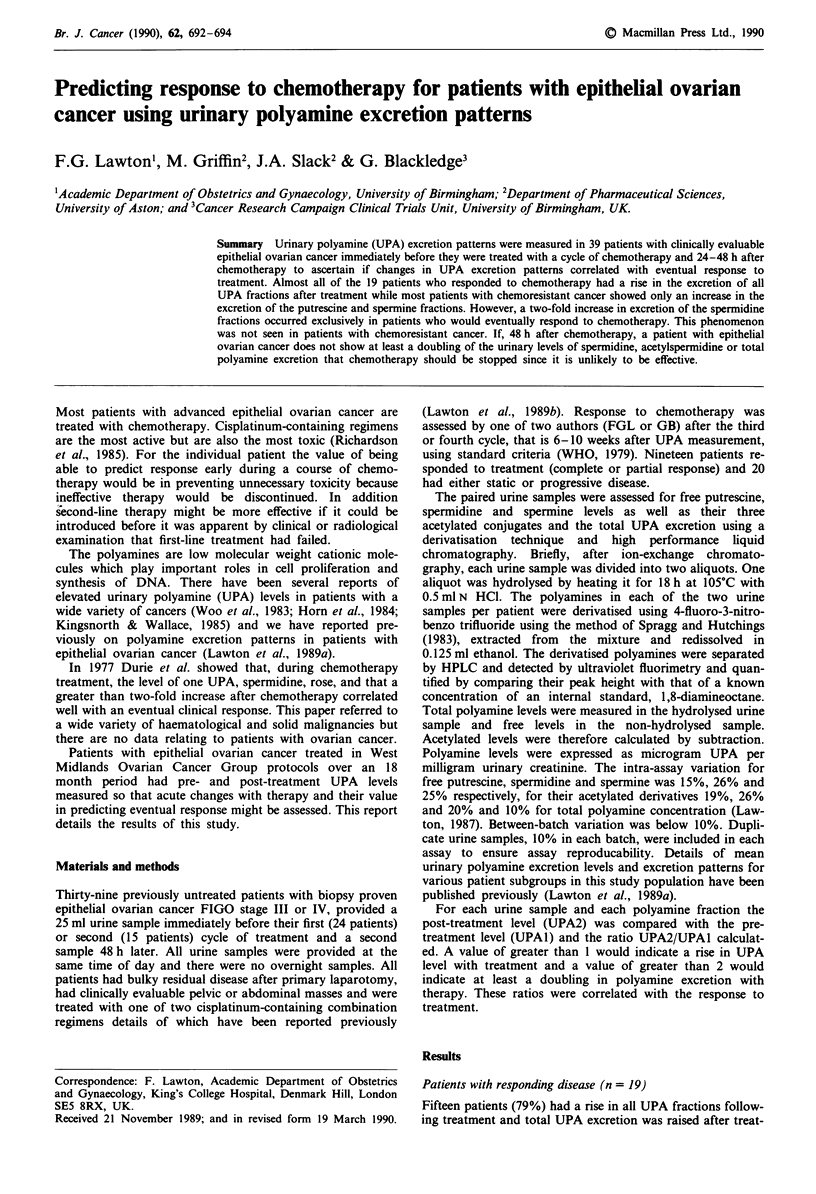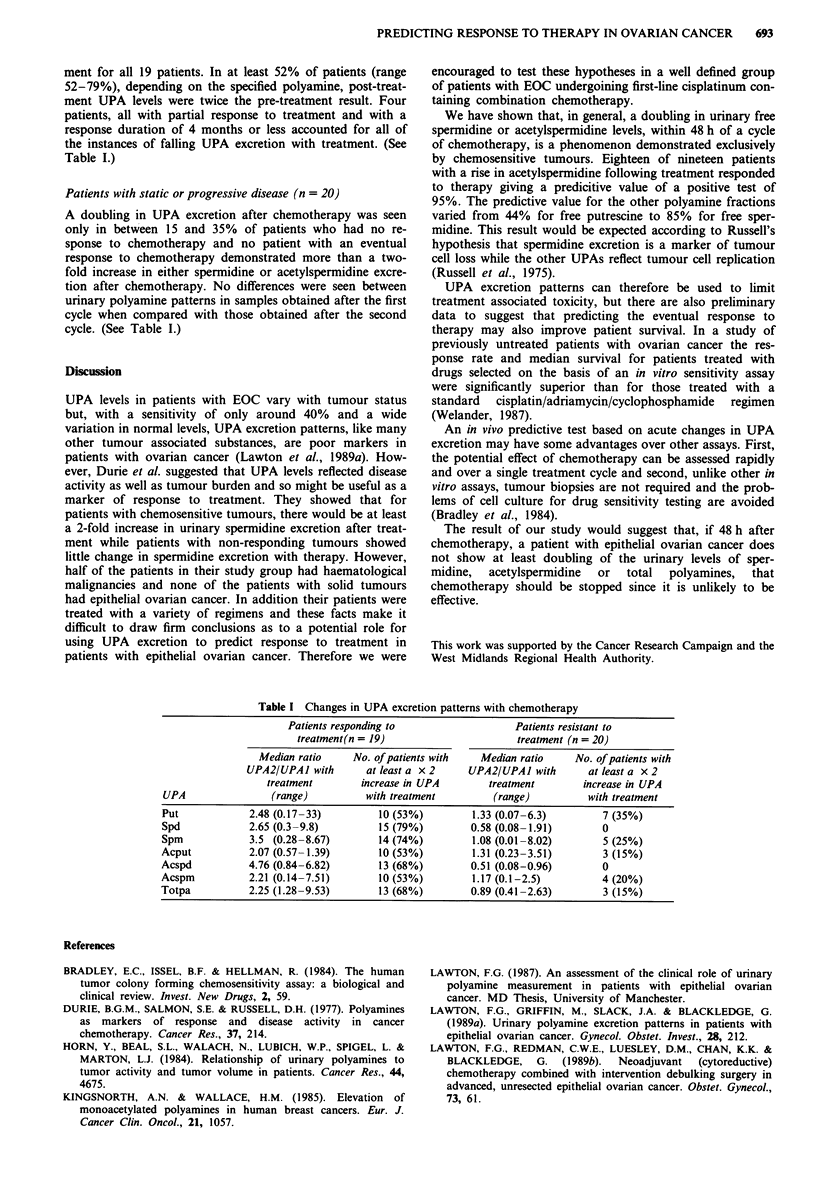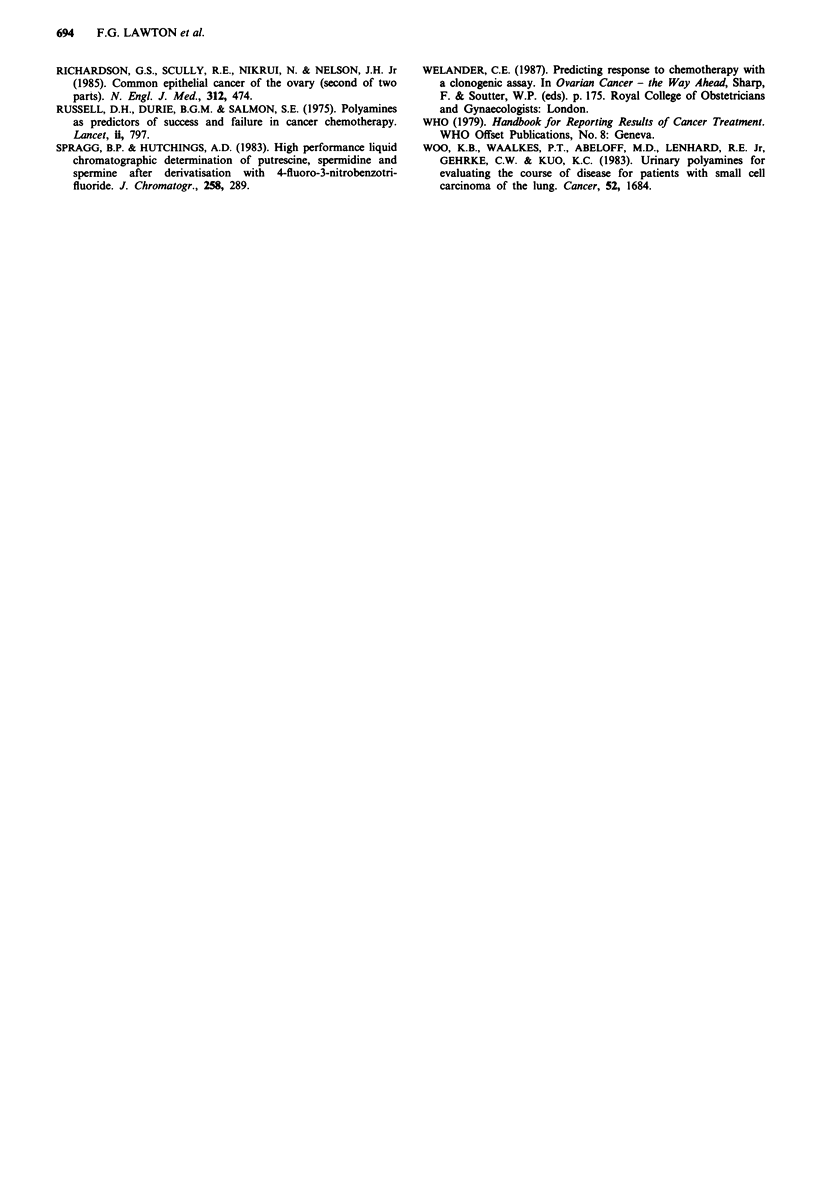# Predicting response to chemotherapy for patients with epithelial ovarian cancer using urinary polyamine excretion patterns.

**DOI:** 10.1038/bjc.1990.359

**Published:** 1990-10

**Authors:** F. G. Lawton, M. Griffin, J. A. Slack, G. Blackledge

**Affiliations:** Academic Department of Obstetrics and Gynaecology, University of Birmingham, UK.

## Abstract

Urinary polyamine (UPA) excretion patterns were measured in 39 patients with clinically evaluable epithelial ovarian cancer immediately before they were treated with a cycle of chemotherapy and 24-48 h after chemotherapy to ascertain if changes in UPA excretion patterns correlated with eventual response to treatment. Almost all of the 19 patients who responded to chemotherapy had a rise in the excretion of all UPA fractions after treatment while most patients with chemoresistant cancer showed only an increase in the excretion of the putrescine and spermine fractions. However, a two-fold increase in excretion of the spermidine fractions occurred exclusively in patients who would eventually respond to chemotherapy. This phenomenon was not seen in patients with chemoresistant cancer. If, 48 h after chemotherapy, a patient with epithelial ovarian cancer does not show at least a doubling of the urinary levels of spermidine, acetylspermidine or total polyamine excretion that chemotherapy should be stopped since it is unlikely to be effective.


Br. J. Cancer (1990), 62, 692-694 © Macmillan Press Ltd., 1990

### Predicting response to chemotherapy for patients with epithelial ovarian cancer using urinary polyamine excretion patterns

F.G. Lawton[1], M. Griffin[2], J.A. Slack[2] & G. Blackledge[3]

[1]*Academic Department of Obstetrics and Gynaecology, University of Birmingham;* [2]*Department of Pharmaceutical Sciences, University of Aston; and* [3]*Cancer Research Campaign Clinical Trials Unit, University of Birmingham, UK.*

**Summary** Urinary polyamine (UPA) excretion patterns were measured in 39 patients with clinically evaluable epithelial ovarian cancer immediately before they were treated with a cycle of chemotherapy and 24-48 h after chemotherapy to ascertain if changes in UPA excretion patterns correlated with eventual response to treatment. Almost all of the 19 patients who responded to chemotherapy had a rise in the excretion of all UPA fractions after treatment while most patients with chemoresistant cancer showed only an increase in the excretion of the putrescine and spermine fractions. However, a two-fold increase in excretion of the spermidine fractions occurred exclusively in patients who would eventually respond to chemotherapy. This phenomenon was not seen in patients with chemoresistant cancer. If, 48 h after chemotherapy, a patient with epithelial ovarian cancer does not show at least a doubling of the urinary levels of spermidine, acetylspermidine or total polyamine excretion that chemotherapy should be stopped since it is unlikely to be effective.

Most patients with advanced epithelial ovarian cancer are treated with chemotherapy. Cisplatinum-containing regimens are the most active but are also the most toxic (Richardson *et al.*, 1985). For the individual patient the value of being able to predict response early during a course of chemotherapy would be in preventing unnecessary toxicity because ineffective therapy would be discontinued. In addition second-line therapy might be more effective if it could be introduced before it was apparent by clinical or radiological examination that first-line treatment had failed.

The polyamines are low molecular weight cationic molecules which play important roles in cell proliferation and synthesis of DNA. There have been several reports of elevated urinary polyamine (UPA) levels in patients with a wide variety of cancers (Woo *et al.*, 1983; Horn *et al.*, 1984; Kingsnorth & Wallace, 1985) and we have reported previously on polyamine excretion patterns in patients with epithelial ovarian cancer (Lawton *et al.*, 1989*a*).

In 1977 Durie *et al.* showed that, during chemotherapy treatment, the level of one UPA, spermidine, rose, and that a greater than two-fold increase after chemotherapy correlated well with an eventual clinical response. This paper referred to a wide variety of haematological and solid malignancies but there are no data relating to patients with ovarian cancer.

Patients with epithelial ovarian cancer treated in West Midlands Ovarian Cancer Group protocols over an 18 month period had pre- and post-treatment UPA levels measured so that acute changes with therapy and their value in predicting eventual response might be assessed. This report details the results of this study.

### Materials and methods

Thirty-nine previously untreated patients with biopsy proven epithelial ovarian cancer FIGO stage III or IV, provided a 25 ml urine sample immediately before their first (24 patients) or second (15 patients) cycle of treatment and a second sample 48 h later. All urine samples were provided at the same time of day and there were no overnight samples. All patients had bulky residual disease after primary laparotomy, had clinically evaluable pelvic or abdominal masses and were treated with one of two cisplatinum-containing combination regimens details of which have been reported previously (Lawton *et al.*, 1989*b*). Response to chemotherapy was assessed by one of two authors (FGL or GB) after the third or fourth cycle, that is 6-10 weeks after UPA measurement, using standard criteria (WHO, 1979). Nineteen patients responded to treatment (complete or partial response) and 20 had either static or progressive disease.

The paired urine samples were assessed for free putrescine, spermidine and spermine levels as well as their three acetylated conjugates and the total UPA excretion using a derivatisation technique and high performance liquid chromatography. Briefly, after ion-exchange chromatography, each urine sample was divided into two aliquots. One aliquot was hydrolysed by heating it for 18 h at 105°C with 0.5 ml N HCl. The polyamines in each of the two urine samples per patient were derivatised using 4-fluoro-3-nitrobenzo trifluoride using the method of Spragg and Hutchings (1983), extracted from the mixture and redissolved in 0.125 ml ethanol. The derivatised polyamines were separated by HPLC and detected by ultraviolet fluorimetry and quantified by comparing their peak height with that of a known concentration of an internal standard, 1,8-diamineoctane. Total polyamine levels were measured in the hydrolysed urine sample and free levels in the non-hydrolysed sample. Acetylated levels were therefore calculated by subtraction. Polyamine levels were expressed as microgram UPA per milligram urinary creatinine. The intra-assay variation for free putrescine, spermidine and spermine was 15%, 26% and 25% respectively, for their acetylated derivatives 19%, 26% and 20% and 10% for total polyamine concentration (Lawton, 1987). Between-batch variation was below 10%. Duplicate urine samples, 10% in each batch, were included in each assay to ensure assay reproducability. Details of mean urinary polyamine excretion levels and excretion patterns for various patient subgroups in this study population have been published previously (Lawton *et al.*, 1989*a*).

For each urine sample and each polyamine fraction the post-treatment level (UPA2) was compared with the pre-treatment level (UPA1) and the ratio UPA2/UPA1 calculated. A value of greater than 1 would indicate a rise in UPA level with treatment and a value of greater than 2 would indicate at least a doubling in polyamine excretion with therapy. These ratios were correlated with the response to treatment.

### Results

### *Patients with responding disease* ($n = 19$)

Fifteen patients (79%) had a rise in all UPA fractions following treatment and total UPA excretion was raised after treat-

Correspondence: F. Lawton, Academic Department of Obstetrics and Gynaecology, King's College Hospital, Denmark Hill, London SE5 8RX, UK.
Received 21 November 1989; and in revised form 19 March 1990.

ment for all 19 patients. In at least 52% of patients (range 52–79%), depending on the specified polyamine, post-treatment UPA levels were twice the pre-treatment result. Four patients, all with partial response to treatment and with a response duration of 4 months or less accounted for all of the instances of falling UPA excretion with treatment. (See Table I.)

*Patients with static or progressive disease (n = 20)*

A doubling in UPA excretion after chemotherapy was seen only in between 15 and 35% of patients who had no response to chemotherapy and no patient with an eventual response to chemotherapy demonstrated more than a two-fold increase in either spermidine or acetylspermidine excretion after chemotherapy. No differences were seen between urinary polyamine patterns in samples obtained after the first cycle when compared with those obtained after the second cycle. (See Table I.)

### Discussion

UPA levels in patients with EOC vary with tumour status but, with a sensitivity of only around 40% and a wide variation in normal levels, UPA excretion patterns, like many other tumour associated substances, are poor markers in patients with ovarian cancer (Lawton *et al.*, 1989*a*). However, Durie *et al.* suggested that UPA levels reflected disease activity as well as tumour burden and so might be useful as a marker of response to treatment. They showed that for patients with chemosensitive tumours, there would be at least a 2-fold increase in urinary spermidine excretion after treatment while patients with non-responding tumours showed little change in spermidine excretion with therapy. However, half of the patients in their study group had haematological malignancies and none of the patients with solid tumours had epithelial ovarian cancer. In addition their patients were treated with a variety of regimens and these facts make it difficult to draw firm conclusions as to a potential role for using UPA excretion to predict response to treatment in patients with epithelial ovarian cancer. Therefore we were encouraged to test these hypotheses in a well defined group of patients with EOC undergoining first-line cisplatinum containing combination chemotherapy.

We have shown that, in general, a doubling in urinary free spermidine or acetylspermidine levels, within 48 h of a cycle of chemotherapy, is a phenomenon demonstrated exclusively by chemosensitive tumours. Eighteen of nineteen patients with a rise in acetylspermidine following treatment responded to therapy giving a predicitive value of a positive test of 95%. The predictive value for the other polyamine fractions varied from 44% for free putrescine to 85% for free spermidine. This result would be expected according to Russell's hypothesis that spermidine excretion is a marker of tumour cell loss while the other UPAs reflect tumour cell replication (Russell *et al.*, 1975).

UPA excretion patterns can therefore be used to limit treatment associated toxicity, but there are also preliminary data to suggest that predicting the eventual response to therapy may also improve patient survival. In a study of previously untreated patients with ovarian cancer the response rate and median survival for patients treated with drugs selected on the basis of an *in vitro* sensitivity assay were significantly superior than for those treated with a standard cisplatin/adriamycin/cyclophosphamide regimen (Welander, 1987).

An *in vivo* predictive test based on acute changes in UPA excretion may have some advantages over other assays. First, the potential effect of chemotherapy can be assessed rapidly and over a single treatment cycle and second, unlike other *in vitro* assays, tumour biopsies are not required and the problems of cell culture for drug sensitivity testing are avoided (Bradley *et al.*, 1984).

The result of our study would suggest that, if 48 h after chemotherapy, a patient with epithelial ovarian cancer does not show at least doubling of the urinary levels of spermidine, acetylspermidine or total polyamines, that chemotherapy should be stopped since it is unlikely to be effective.

This work was supported by the Cancer Research Campaign and the West Midlands Regional Health Authority.

**Table I** Changes in UPA excretion patterns with chemotherapy

| | *Patients responding to treatment(n = 19)* | | *Patients resistant to treatment (n = 20)* | |
|---|---|---|---|---|
| *UPA* | *Median ratio UPA2/UPA1 with treatment (range)* | *No. of patients with at least a × 2 increase in UPA with treatment* | *Median ratio UPA2/UPA1 with treatment (range)* | *No. of patients with at least a × 2 increase in UPA with treatment* |
| Put | 2.48 (0.17–33) | 10 (53%) | 1.33 (0.07–6.3) | 7 (35%) |
| Spd | 2.65 (0.3–9.8) | 15 (79%) | 0.58 (0.08–1.91) | 0 |
| Spm | 3.5 (0.28–8.67) | 14 (74%) | 1.08 (0.01–8.02) | 5 (25%) |
| Acput | 2.07 (0.57–1.39) | 10 (53%) | 1.31 (0.23–3.51) | 3 (15%) |
| Acspd | 4.76 (0.84–6.82) | 13 (68%) | 0.51 (0.08–0.96) | 0 |
| Acspm | 2.21 (0.14–7.51) | 10 (53%) | 1.17 (0.1–2.5) | 4 (20%) |
| Totpa | 2.25 (1.28–9.53) | 13 (68%) | 0.89 (0.41–2.63) | 3 (15%) |

### References

BRADLEY, E.C., ISSEL, B.F. & HELLMAN, R. (1984). The human tumor colony forming chemosensitivity assay: a biological and clinical review. *Invest. New Drugs*, **2**, 59.

DURIE, B.G.M., SALMON, S.E. & RUSSELL, D.H. (1977). Polyamines as markers of response and disease activity in cancer chemotherapy. *Cancer Res.*, **37**, 214.

HORN, Y., BEAL, S.L., WALACH, N., LUBICH, W.P., SPIGEL, L. & MARTON, L.J. (1984). Relationship of urinary polyamines to tumor activity and tumor volume in patients. *Cancer Res.*, **44**, 4675.

KINGSNORTH, A.N. & WALLACE, H.M. (1985). Elevation of monoacetylated polyamines in human breast cancers. *Eur. J. Cancer Clin. Oncol.*, **21**, 1057.

LAWTON, F.G. (1987). An assessment of the clinical role of urinary polyamine measurement in patients with epithelial ovarian cancer. MD Thesis, University of Manchester.

LAWTON, F.G., GRIFFIN, M., SLACK, J.A. & BLACKLEDGE, G. (1989*a*). Urinary polyamine excretion patterns in patients with epithelial ovarian cancer. *Gynecol. Obstet. Invest.*, **28**, 212.

LAWTON, F.G., REDMAN, C.W.E., LUESLEY, D.M., CHAN, K.K. & BLACKLEDGE, G. (1989*b*). Neoadjuvant (cytoreductive) chemotherapy combined with intervention debulking surgery in advanced, unresected epithelial ovarian cancer. *Obstet. Gynecol.*, **73**, 61.

RICHARDSON, G.S., SCULLY, R.E., NIKRUI, N. & NELSON, J.H. Jr (1985). Common epithelial cancer of the ovary (second of two parts). *N. Engl. J. Med.*, **312**, 474.

RUSSELL, D.H., DURIE, B.G.M. & SALMON, S.E. (1975). Polyamines as predictors of success and failure in cancer chemotherapy. *Lancet*, **ii**, 797.

SPRAGG, B.P. & HUTCHINGS, A.D. (1983). High performance liquid chromatographic determination of putrescine, spermidine and spermine after derivatisation with 4-fluoro-3-nitrobenzotrifluoride. *J. Chromatogr.*, **258**, 289.

WELANDER, C.E. (1987). Predicting response to chemotherapy with a clonogenic assay. In *Ovarian Cancer – the Way Ahead*, Sharp, F. & Soutter, W.P. (eds). p. 175. Royal College of Obstetricians and Gynaecologists: London.

WHO (1979). *Handbook for Reporting Results of Cancer Treatment*. WHO Offset Publications, No. 8: Geneva.

WOO, K.B., WAALKES, P.T., ABELOFF, M.D., LENHARD, R.E. Jr, GEHRKE, C.W. & KUO, K.C. (1983). Urinary polyamines for evaluating the course of disease for patients with small cell carcinoma of the lung. *Cancer*, **52**, 1684.